# Measuring and Targeting Persistent Inflammation in Chronic Coronary Disease

**DOI:** 10.1016/j.jacadv.2024.100997

**Published:** 2024-06-04

**Authors:** Nikolaos G. Frangogiannis

**Affiliations:** Department of Medicine (Cardiology), The Wilf Family Cardiovascular Research Institute, Albert Einstein College of Medicine, Bronx, New York, USA

**Keywords:** coronary disease, inflammation, statins

**Figure:**
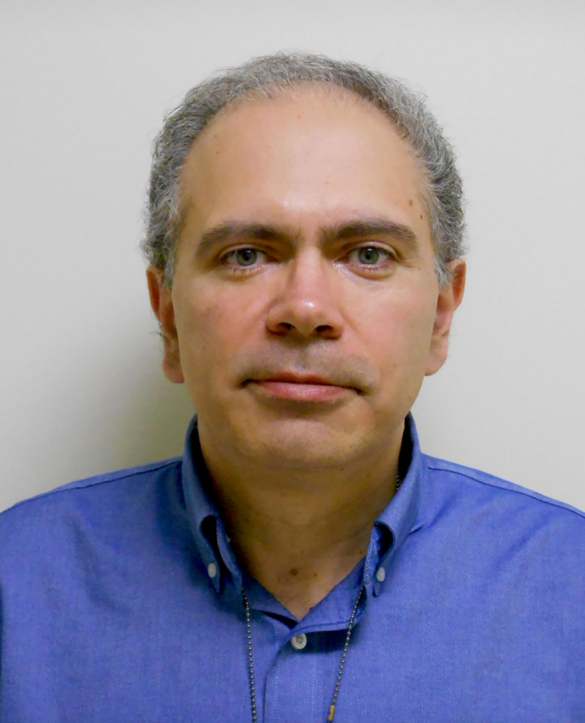

Lowering low-density lipoprotein-cholesterol (LDL-C) is the most effective therapeutic strategy for the prevention of cardiovascular events in patients with chronic atherosclerotic coronary artery disease (CAD). Several clinical trials have demonstrated that aggressive treatment with statins significantly reduces morbidity and mortality in CAD patients, supporting the notion that for LDL-C levels, “lower is better.” However, despite the great success of statins in improving outcome and the effectiveness of other adjunctive LDL-C lowering interventions (such as ezetimibe and PCSK9 inhibitors), a large number of chronic CAD patients remain at risk for adverse events. Although extensive evidence suggests that persistent low-grade vascular inflammation may contribute to the pathogenesis of atherosclerotic coronary disease, the prognostic contribution of inflammatory risk in CAD patients remains poorly defined. Considering that statins are potent suppressors of vascular inflammation, what is the prognostic significance of persistent low-grade inflammation in patients with chronic CAD receiving guideline-directed medical care?

The REAL-CAD (Randomized Evaluation of Aggressive or Moderate Lipid Lowering Therapy with Pitavastatin in Coronary Artery Disease) trial demonstrated that high-dose pitavastatin (4 mg/d) reduced cardiovascular events in Japanese patients with chronic stable CAD, when compared with low-dose pitavastatin (1 mg/d), thus supporting the superiority of aggressive statin therapy.^1^ In this issue of *JACC: Advances*, Iwata et al^2^ report findings from a prespecified subanalysis of the REAL-CAD trial, aimed to assess the prognostic impact of persistent low-grade inflammation (defined by high-sensitivity C-reactive protein (hsCRP) levels above the median 0.5 mg/L) in statin-treated patients with chronic CAD. The authors found that patients with persistent elevation of hsCRP had higher cardiovascular mortality and an increased incidence of events in comparison to the group with persistently low hsCRP levels. The detrimental effect of persistent inflammation was independent of baseline and achieved LDL-C levels in statin-treated patients. It should be emphasized that the median hsCRP levels in the current study were at least 4 times lower than the corresponding levels in published studies examining the effects of anti-inflammatory strategies in patients with CAD (0.5 mg/L in the REAL-CAD trial vs >2 mg/L in most other studies).^3^^,^^4^ Based on the findings by Iwata et al, should we redefine the use of hsCRP levels as a marker of significant residual inflammatory risk to include individuals with hsCRP levels <2 mg/L?

## The use of hsCRP levels to assess inflammatory risk

hsCRP has been extensively used to assess inflammatory risk in patients with chronic CAD; however, a consensus regarding the clinical significance of the levels is lacking. Although in the REAL-CAD study, Iwata et al observed significant inflammatory risk in Japanese patients at hsCRP levels much lower than other studies, the significance of hsCRP levels <1 mg/L remains unknown. The findings may reflect diverse responses to inflammatory injury in various races and ethnicities. Until large studies establish race-specific cutoffs, hsCRP levels >2 mg/L should be considered as evidence of persistent vascular inflammation associated with increased cardiovascular risk.^5^

## Are hsCRP levels sufficient for assessment of inflammatory risk in CAD patients?

Inflammation encompasses a broad range of cellular responses that involve immune cells, vascular cells, and organ parenchymal cells, and cannot be quantitatively measured by a single marker. In addition to hsCRP, several other biomarkers have been tested in chronic CAD patients and may provide independent prognostic information. For example, neutrophil-derived indicators were found to predict all-cause mortality in CAD patients at low residual inflammatory risk, independently of hsCRP levels.^6^ Moreover, in a comparison of interleukin (IL)-6, LDL-C and hsCRP in CAD patients enrolled in the Cardiovascular Inflammation Reduction Trial, IL-6 levels were the strongest predictor of all-cause mortality.^7^ In addition to more specific serum biomarkers, emerging molecular imaging modalities can contribute information on the inflammatory profile of the plaques, thus identifying patients with high-risk lesions that may benefit from anti-inflammatory interventions.^8^ Although new more specific and pathophysiologically relevant biomarkers and imaging strategies may eventually prove valuable, successful implementation of anti-inflammatory approaches in the clinical setting requires well-validated, simple, practical, and inexpensive markers. Thus, despite its lack of specificity, hsCRP remains the most reliable, informative, and widely used tool for inflammatory risk assessment in chronic CAD patients.

## Therapeutic options for patients with increased inflammatory risk

Despite decades of intense and highly productive research on pathway-specific anti-inflammatory therapeutics, it was an ancient drug with pleiotropic effects that provided the first hope for implementation of an anti-inflammatory strategy in patients with chronic CAD. Colchicine, an inhibitor of tubulin polymerization, exerts a broad range of anti-inflammatory actions, perturbing neutrophil migration, adhesion and activation, and inhibiting NLRP3 inflammasome activity.^9^ In patients with chronic CAD, low-dose colchicine was found to decrease hsCRP levels and reduce the incidence of major cardiovascular events by 31%,^4^ without any effects on all-cause mortality. Although hsCRP levels were not an entry criterion in the major colchicine trials, CAD patients with residual inflammatory risk evidenced by elevated hsCRP levels may benefit from low-dose colchicine administration. More specific anti-cytokine approaches may also hold promise. In CANTOS (Canakinumab Anti-Inflammatory Thrombosis Outcomes Study), treatment of patients with prior myocardial infarction and high hsCRP levels with canakinumab, an antibody against the potent pro-inflammatory cytokine IL-1β, reduced the risk of the composite endpoint (death, nonfatal myocardial infarction, or nonfatal stroke) by 15% in comparison to guideline-directed treatment.^3^ Approaches inhibiting other inflammatory cytokines (such as IL-6) are currently being tested. Additional strategies that suppress inflammation by targeting neutrophils, by disrupting chemokine-stimulated inflammatory signaling, or macrophage activation,^10^ or by administering anti-inflammatory pro-resolving lipid mediators^11^ may hold promise for future clinical investigations.

It should be emphasized that chronic treatment with anti-inflammatory mediators in CAD patients may carry significant risks, caused by perturbations in host defense mechanisms. Chronic inhibition of IL-1β in the CANTOS trial was associated with a higher incidence of fatal infections and sepsis. Other interventions targeting immune pathways may have an impact on tumorigenesis or on tissue repair. Thus, identification of anti-inflammatory agents with favorable safety records is a priority. The extensive use of selective anti-inflammatory agents in rheumatologic disorders provides a valuable body of experience for selection of relatively safe candidates that can be tested as inhibitors of persistent low-grade vascular inflammation in chronic CAD patients.

## Funding support and author disclosures

Dr Frangogiannis’ laboratory is supported by 10.13039/100000002NIH grants R01 HL76246, R01 HL85440, and R01 HL149407, and by 10.13039/100000005Department of Defense grant PR211352.
